# The course of headache in patients with moderate-to-severe headache due to mild traumatic brain injury: a retrospective cross-sectional study

**DOI:** 10.1186/s10194-017-0755-9

**Published:** 2017-04-20

**Authors:** Chang-Ki Hong, Jin-Yang Joo, Yu Shik Shim, Sook Young Sim, Min A Kwon, Yong Bae Kim, Joonho Chung

**Affiliations:** 1 Department of Neurosurgery, Gangnam Severance Hospital, Yonsei University College of Medicine, 211, Eonjuro, Gangnam-gu, Seoul, 135-720 Republic of Korea; 2 Severance Institute for Vascular and Metabolic Research, Yonsei University, Seoul, Republic of Korea; 3 Department of Neurosurgery, Inha University School of Medicine and Hospital, Incheon, Republic of Korea; 4 Department of Neurosurgery, Inje University Seoul Paik Hospital, Seoul, Republic of Korea; 5 Department of Neurological Surgery, Rush University Medical Center, 1725 W. Harrison St., Professional Building Suite 855, Chicago, IL 60612 USA

**Keywords:** Headache, Head injury, Post-traumatic headache, Traumatic brain injury

## Abstract

**Background:**

Little is known about the long-term course of headache in patients with moderate-to-severe headache due to traumatic brain injury ﻿(TBI). We evaluated the course of headache in patients with moderate-to-severe headache due to mild TBI.

**Methods:**

Since September 2009, patients with TBI prospectively rated their headache using a numeric rating scale (NRS). From the database containing 935 patients with TBI between September 2009 and December 2013, 259 patients were included according to following criteria: (1) newly onset moderate-to-severe headache (NRS ≥ 4) due to head trauma; (2) age ≥ 15 years; (3) Glasgow Coma Scale ≥ 13; (4) transient loss of consciousness ≤ 30 min; and (5) radiographic evaluation, such as computed tomography or magnetic resonance image. We evaluated initial and follow-up NRS scores to determine the significance of NRS changes and identified risk factors for moderate-to-severe headache at 36-month follow-up.

**Results:**

At 36-month follow-up, 225 patients (86.9%) reported improved headache (NRS ≤ 3) while 34 (13.1%) reported no improvement. The NRS scores were significantly decreased within a month (*P* < 0.001). The follow-up NRS scores at 12-, 24-, and 36-months were lower than those at one month (*P* < 0.001). Multiple logistic regression analysis showed that post-traumatic seizure (odds ratio, 2.162; 95% CI, 1.095–6.542; *P* = 0.041) and traumatic intracranial hemorrhage (odds ratio, 2.854; 95% CI, 1.241–10.372; *P* = 0.024) were independent risk factors for moderate-to-severe headache at 36-month follow-up.

**Conclusions:**

The course of headache in patients with mild TBI continuously improved until 36-month follow-up. However, 13.1% of patients still suffered from moderate-to-severe headache at 36-month follow-up, for whom post-traumatic seizure and traumatic intracranial hemorrhage might be risk factors.

## Background

It has been reported that 18–33% of subjects continue to experience persistent headache after traumatic brain injury (TBI) even beyond one year post-injury. Most headaches after TBI resolves within 6–12 months [1]. Previously, studies that addressed headache in patients with TBI received little attention since achieving a good clinical outcome was of higher importance than headache improvement. However, headache has become one of the most frequently reported symptoms after mild TBI [1–3] and has affected quality of life [4]. Thus, persistent post-traumatic headache, rather than some mild morbidity after mild TBI, has received more attention because of its potential impact on patient quality of life. According to the International Classification of Headache Disorders, 3^rd^ edition (beta version), headache attributed to trauma or injury to the head and/or neck are the most common secondary headache disorders [5]. Post-traumatic headache is reported to have developed within 7 days after one of the following: (1) the injury to the head, (2) regaining of consciousness following the injury to the head, or (3) discontinuation of medications that impair ability to sense or report headache following the injury to the head [5]. During the first 3 months from onset, post-traumatic headache is considered acute. Persistent post-traumatic headache was described as headache of greater than 3 months’ duration caused by traumatic injury to the head. The term persistent has been adopted in place of chronic [5]. However, little is known about how these headaches change over time and what makes these headaches persistent. Thus, it is important to know about the course of headache after mild TBI. The purpose of this study was to evaluate the courses of headache in patients who had moderate-to-severe headache initially after mild TBI and to identify associated risk factors.

## Methods

This retrospective cross-sectional study using prospectively collected data was approved by our institutional review board, and informed consent was waived. Since September 2009, we prospectively rated patient headache using a numeric rating scale (NRS). From the database containing 935 patients with TBI between September 2009 and December 2013, 259 patients were included in the present study according to the following inclusion criteria: (1) newly onset moderate-to-severe headache (NRS ≥ 4) due to head trauma; (2) age ≥ 15 years; (3) Glasgow Coma Scale [GCS] ≥ 13; (4) transient loss of consciousness (LOC) ≤ 30 min; and (5) radiographic evaluation, such as computed tomography (CT) or magnetic resonance image (MRI). The remaining 676 case from the database were excluded for the following reasons: (1) GCS < 13 (*n* = 228); (2) initial or follow-up modified Rankin Scale (mRS) ≥ 3 (*n* = 59); (3) history of neurosurgery (craniotomy due to any cause) (*n* = 37); (4) TBI due to any cerebrovascular disease (*n* = 87); (5) history of previously diagnosed intracranial disease (*n* = 45); (6) post-traumatic amnesia > 24 h (*n* = 33); (7) delayed hemorrhage (>24 h) (*n* = 25); (8) combined spinal cord injury (*n* = 93); (9) incomplete NRS score data (*n* = 17); and (10) loss to follow-up (*n* = 52). Our inclusion/exclusion criteria have contained the definition of mild TBI since mild TBI, in general, is defined as TBI resulting in GCS ≥ 13, LOC ≤ 30 min, and post-traumatic amnesia ≤ 24 h [6, 7]. Most mild TBI are not associated with visible abnormalities on structural neuroimaging. A complicated mild TBI was differentiated from an uncomplicated mild TBI by the presence of (a) a depressed skull fracture and/or (b) a trauma-related intracranial abnormality, such as hemorrhage, contusion, or edema [8]. According to the GCS scoring system, moderate TBI is defined as a GCS of 9 to 12 and severe TBI is defined as a GCS of 3 to 8.

Intensity of headache was assessed with the NRS, an 11-point numeric scale for rating pain intensity. The quantitative scale ranges from 0 to 10, with 0 meaning “no headache at all” and 10 meaning “the worst possible headache.” This 11-point scale has been previously applied to headache assessment [9–11]. We defined NRS scores of 1 to 3, 4 to 6, and 7 to 10 as mild, moderate, and severe headaches, respectively. Mild headache was described as nagging, annoying, or interfering little with active daily living. Moderate headache was described as interfering significantly with active daily living. Severe headache was described as disabling or interfering to the point that patients were unable to perform daily activities. Thus, we defined an NRS score ≥ 4 as significant. All included patients visited the emergency department or the outpatient office within 48 h of onset. NRS score was determined every 8 h (three times per day) during a patient’s hospitalization or twice during each outpatient visit. Analgesics were prescribed to all included patients. Patient NRS scores were collected and averaged for the onset (within a week from TBI), and at 1-month, 6-month, 12-month, 24-month, and 36-month follow-ups.

Three independent investigators blinded to the data retrospectively reviewed the clinical and radiographic data of the included patients using their medical records. Variables evaluated were age, gender, initial GCS score, cause of TBI, mechanism of TBI, traumatic intracranial hemorrhage, initial symptoms and signs (LOC, vomiting, tinnitus, visual symptoms, confusion, post-traumatic amnesia, and post-traumatic seizure), and previous physician-diagnosed headache (treated with medications). A follow-up NRS score ≤ 3 (mild headache or no headache) was defined as improvement, as NRS scores rated 1 to 3 were regarded as headache interfering minimally with most activities [11]. We evaluated the initial and follow-up NRS scores to determine the significance of NRS changes and identified risk factors for moderate-to-severe headache (NRS ≥ 4) at 36-month follow-up.

### Statistical analysis

All statistical analyses were performed in consultation with a biostatistician and performed using R language ver. 3.01 (R Foundation for Statistical Computing, Vienna, Austria). Paired *t*-tests or Wilcoxon signed rank tests were used to evaluate NRS changes between baseline and follow-up scores. For these multiple comparisons, we applied the Bonferroni adjustment to the interpretations; therefore, statistical significance was defined by *P* < 0.01 (0.05/5). Mann-Whitney *U-*tests were used for numeric variables. Chi-square tests or Fisher’s exact tests were used for nominal variables. Logistic regression analysis was performed to determine the independent risk factors of moderate-to-severe headache at 36-month follow-up (with regard to NRS score change). Multiple logistic regression analysis was performed on variables with an unadjusted effect and a *P* < 0.1 on simple logistic regression analysis. Statistical significance was defined as *P* < 0.05 with a 95% confidence interval (CI).

## Results

Table 1 presents patient clinical characteristics. On admission, the most frequent cause of TBI was motor vehicle accident (142 patients, 54.8%) and the most frequent mechanism was head striking an object (170 patients, 65.6%). LOC was experienced in 144 patients (55.6%), post-traumatic amnesia in 57 patients (22.0%), post-traumatic seizure in 16 patients (6.2%), traumatic intracranial hemorrhage in 42 patients (16.2%), and previously physician-diagnosed headache in 52 patients (20.1%). Forty-two patients with traumatic intracranial hemorrhage had neurologically asymptomatic minimal hemorrhage on their CT or MRI. At 12-month follow-up, 154 patients (59.5%) improved their headache (NRS ≤ 3) and 105 patients (40.5%) still suffered from moderate-to-severe headache. There were no significant differences in characteristics between patients with headache improvement and those without headache improvement.

**Table 1:** Clinical Characteristics of the patients

	Baseline characteristics (*n* = 259)	12-month follow-up			36-month follow-up		
		Headache improved (*n* = 154)	Headache not improved (*n* = 105)	*P* value	Headache improved (*n* = 225)	Headache not improved (*n* = 34)	*P* value
Age (mean ± SD)	37.9 ± 10.4	39.4 ± 11.4	38.6 ± 12.1	0.801	40.4 ± 12.5	42.1 ± 12.8	0.545
Female (n, %)	116 (44.8)	72 (46.8)	44 (41.9)	0.685	102 (45.3)	14 (41.2)	0.617
Cause of traumatic brain injury (n, %)				0.651			0.452
Motor vehicle accident	142 (54.8)	93 (60.4)	59 (56.2)		122 (54.2)	20 (58.8)	
Fall from height	61 (23.6)	33 (21.4)	28 (26.7)		57 (25.3)	4 (11.8)	
Direct hit on the head	56 (21.6)	28 (18.2)	28 (26.7)		46 (20.4)	10 (29.4)	
Mechanism of traumatic brain injury (n, %)				0.434			0.885
Whiplash	36 (13.9)	22 (14.3)	14 (13.3)		31 (13.8)	5 (14.7)	
Head being struck	53 (20.5)	33 (21.4)	20 (19.0)		45 (20.0)	8 (23.5)	
Head striking an object	170 (65.6)	99 (64.3)	71 (67.6)		149 (66.2)	21 (61.8)	
Loss of consciousness (n, %)	144 (55.6)	79 (51.3)	65 (61.9)	0.148	123 (54.7)	21 (61.8)	0.264
Vomiting (n, %)	73 (28.2)	41 (26.6)	32 (20.8)	0.353	66 (29.3)	7 (20.6)	0.192
Tinnitus (n, %)	25 (9.7)	16 (10.4)	7 (8.6)	0.638	23 (10.2)	2 (5.9)	0.312
Visual symptoms (n, %)	18 (6.9)	12 (7.8)	6 (5.7)	0.755	16 (7.1)	2 (5.9)	0.863
Confusion (n, %)	50 (19.3)	32 (20.8)	18 (17.1)	0.621	41 (18.2)	9 (26.5)	0.206
Post-traumatic amnesia (n, %)	57 (22.0)	38 (24.7)	19 (18.1)	0.446	47 (20.9)	10 (29.4)	0.127
Post-traumatic seizure (n, %)	16 (6.2)	3 (1.9)	13 (12.4)	0.145	6 (2.7)	10 (29.4)	0.041
Traumatic intracranial hemorrhage (n, %)	42 (16.2)	10 (6.5)	32 (20.8)	0.072	19 (8.4)	23 (67.6)	0.008
Previously diagnosed headache (n, %)	52 (20.1)	25 (16.2)	27 (25.7)	0.096	41 (18.2)	11 (32.4)	0.076
Hypertension (n, %)	110 (42.5)	71 (46.1)	39 (37.1)	0.185	96 (42.7)	14 (41.2)	0.814
Diabetes (n, %)	51 (19.7)	33 (21.4)	18 (17.1)	0.549	44 (19.6)	7 (20.6)	0.905
Dyslipidemia (n, %)	59 (22.8)	41 (26.6)	18 (17.1)	0.177	54 (24.0)	5 (14.7)	0.258
Smoking (n, %)	76 (29.3)	43 (27.9)	33 (31.4)	0.646	65 (28.9)	11 (32.4)	0.649
Alcohol (n, %)	63 (24.3)	42 (27.3)	21 (20.0)	0.225	56 (24.9)	7 (20.6)	0.561
Antiplatelet agent (n, %)	21 (8.1)	15 (9.7)	6 (5.7)	0.376	19 (8.4)	2 (5.9)	0.454
Anticoagulant (n, %)	5 (1.9)	3 (1.9)	2 (1.9)	0.889	5 (2.2)	0 (0.0)	0.623

*SD* standard deviation

At the 36-month follow-up, 225 patients (86.9%) reported their headache improved and 34 patients (13.1%) still suffered from moderate-to-severe headache. Four patients of the 154 patients with headache improvement at 12-month follow-up reported aggravated headache (NRS ≥ 4) at 36-month follow-up. Seventy-five patients of the 105 patients without headache improvement at 12-month follow-up became better (NRS ≤ 3) at 36-month follow-up. Post-traumatic seizure (*P* = 0.041) and traumatic intracranial hemorrhage (*P* = 0.008) were significantly different between patients with headache improvement and those without headache improvement.

**Fig. 1:**
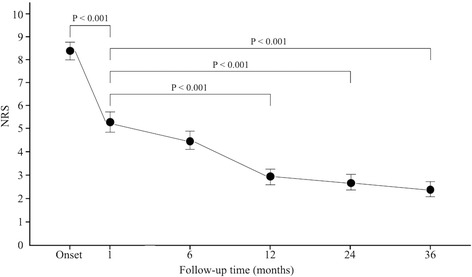
The course of headache in patients with mild traumatic brain injury is represented by changes in numeric rating scale (NRS) score from onset to 36-month follow-up. Compared with NRS scores at onset, those at 1-month follow-up were significantly decreased. Follow-up NRS scores were significantly lower at 12-, 24-, and 36-month follow-ups compared to 1-month follow-up. The NRS scores decreased continuously until 36-month follow-up. *Dots* indicate mean NRS scores at each time point and *error bars* indicate the 95% confidence intervals

Mean NRS scores were 8.37, 5.23, 2.94, 2.62, and 2.35 at onset, 1-month, 12-month, 24-month, and 36-month follow-ups, respectively. The NRS scores were significantly decreased within a month (*P* < 0.001). Then, the follow-up NRS scores at 12-, 24-, and 36-months were lower than those at one month (*P* < 0.001). Follow-up NRS scores decreased continuously until the 36-month follow-up (Fig. 1).

Multiple logistic regression analysis showed that post-traumatic seizure (odds ratio, 2.162; 95% CI, 1.095–6.542; *P* = 0.041) and traumatic intracranial hemorrhage (odds ratio, 2.854; 95% CI, 1.241–10.372; *P* = 0.024) were independent risk factors for moderate-to-severe headache at the 36-month follow-up (Table 2). Previously diagnosed headache had a *P*-value of 0.046 in simple logistic regression analysis, but was not statistically significant after adjustment (*P* = 0.097).

**Table 2:** Risk factors for moderate-to-severe headache at the 36-month follow-up

	Unadjusted		Adjusted	
	OR (95% CI)	*p* value	OR (95% CI)	*p* value
Age in years				
< 40	1		1	
≥ 40	1.628 (0.636–3.743)	0.708	1.446 (0.423–3.190)	0.845
Gender				
Female	1		1	
Male	0.954 (0.438–2.285)	0.713	0.912 (0.497–2.559)	0.776
Initial GCS				
15	1			
13 and 14	1.898 (0.845–5.562)	0.294		
Cause of traumatic brain injury				
Motor vehicle accident	1			
Fall from height	0.733 (0.433–2.632)	0.535		
Direct hit on the head	1.865 (0.524–3.356)	0.629		
Mechanism of traumatic brain injury				
Whiplash	1			
Head being struck	1.438 (0.754–4.224)	0.472		
Head striking an object	0.872 (0.637–3.325)	0.486		
Loss of conscious				
No	1			
Yes	2.762 (0.805–6.433)	0.154		
Vomiting				
No	1			
Yes	0.949 (0.475–1.768)	0.512		
Tinnitus				
No	1			
Yes	0.886 (0.396–2.267)	0.564		
Visual symptoms				
No	1			
Yes	0.821 (0.358–1.947)	0.607		
Confusion				
No	1			
Yes	1.504 (0.526–3.435)	0.658		
Post-traumatic amnesia				
No	1		1	
Yes	2.083 (0.889–6.219)	0.069	1.921 (0.816–4.945)	0.154
Post-traumatic seizure				
No	1		1	
Yes	2.435 (1.218–6.105)	0.032	2.162 (1.095–6.542)	0.041
Traumatic intracranial hemorrhage				
No	1		1	
Yes	3.595 (1.329–9.247)	0.008	2.854 (1.241–10.372)	0.024
Previously diagnosed headache				
No	1		1	
Yes	1.921 (1.056–4.247)	0.046	1.677 (0.896–5.331)	0.097

*CI* confidence interval, *OR* odds ratio

## Discussion

The course of headache in patients with moderate-to-severe headache due to mild TBI continuously improved until 36-month follow-up. However, persistence of moderate-to-severe headache occurred in 13.1% of the 259 patients throughout the 36-month follow-up. Patients who experienced post-traumatic seizure and those who had traumatic intracranial hemorrhage on their radiographic images might be expected to have persistent moderate-to-severe headache at 36-month follow-up.

The reported prevalence of headache due to TBI ranges between 20 and 89% at baseline, and the cumulative incidence of headache over one year has been reported to be 71–91%. Many studies have reported that more than one-third of patients complained of persistent headache throughout the follow-up period [1, 3, 12–16]. The present study is different from previous reports because we included selective patients who had moderate-to-severe headache due to TBI. Thus, it was difficult to assess the prevalence of new onset post-traumatic headache throughout the follow-up period. It was only possible to determine if headache intensity became better or worse over time. Four of the 154 patients whose headache was better at 12-month follow-up might be considered to have new onset post-traumatic headache or headache unrelated to TBI because their headache became better but was then aggravated again at 36-month follow-up. All included patients had follow-up NRS scores that were continuously decreased until 36-month follow-up, except 34 patients (13.1%) who suffered from moderate-to-severe headache over the entire 36 months after TBI. Among those 34 patients, however, their headaches might not have originated from TBI because we did not evaluate NRS continuously and there were time intervals between the follow-up periods. These headaches might be new onset migraines because the prevalence of migraine with severe headache has been reported in 14.2% of Americans and 8.4% of Japanese annually [17, 18]. The characteristics and pathophysiologic mechanisms are similar between post-traumatic headache and migraine [19–22]. In addition, there are no specific headache features known to distinguish the subtypes of headache attributed to trauma or injury to the head and/or neck from other headache types; most often these resemble tension-type headache or migraine [5].

We included patients with moderate-to-severe headache initially, who did not have neurological disability with GCS ≥ 13 because disability could affect post-traumatic headache. In fact, it was reported that disability related to post-traumatic headache was high with an average NRS ranging from 5.5 at baseline to 5.7 at 60-month follow-up [14]. Moderate-to-severe TBI makes post-traumatic headache worse compared to mild TBI [23]. However, we did not focus on the intensity (mild, moderate, or severe) of TBI, but just on moderate-to-severe headache over time in neurologically asymptomatic patients who have frequently received little attention since their clinical course was mostly good. Additionally, moderate-to-severe headache (NRS ≥ 4) might have more clinical relevance compared to mild headache (NRS ≤ 3), because mild headache was described as interfering little with activities of daily living, while moderate-to-severe headache interfered with or prevented activities of daily living. We, also, excluded patients with spinal cord injury because some patients with mild TBI could have headaches secondary to spinal cord injury. In the same context, 55.6% (144/259) of LOC in the present study, that seemed a bit high for patients with mild TBI, can be understood. The initial impact to the head could be more severe in patients of the present study than those with mild TBI in general because our patients had moderate-to-severe headache initially as well as visible abnormalities on structural neuroimaging. In addition, Beetar et al. have previously reported that 62.9% (127/202) of their patients with mild TBI had LOC [24].

We identified two risk factors, post-traumatic seizure and traumatic intracranial hemorrhage, that were related to moderate-to-severe headache at 36-month follow-up. Because our data included patients with traumatic intracranial hemorrhage, we did enroll patients with complicated mild TBI. However, we excluded patients with disability (mRS ≥ 3) because we had tried to get rid of probable effects of disability on the course of headache in patients with TBI. Neurological deficits may affect more seriously to patients’ daily living or quality of life than post-traumatic headache. Thus, our findings on risk factors for moderate-to-severe headache at 36-month follow-up might be natural for patients with complicated mild TBI because post-traumatic seizure and traumatic intracranial hemorrhage usually occur in patients with complicated mild TBI compared to patients with uncomplicated mild TBI. Up to 3 years after TBI, however, patients could have post-traumatic headache that interfered with or prevented activities of daily living according to our results. These results may allow providers to educate their patients who present with initially alert consciousness and no disability about what they can expect with respect to moderate-to-severe headache after TBI. Additionally, this suggests that management of post-traumatic headache should not be neglected.

There are several limitations to this study. First, it was a retrospective study based on a prospectively collected database and had no true control group. Patients with incomplete NRS data (*n* = 17) and those lost to follow-up (*n* = 52) were excluded from the analyses. Second, we did not distinguish headache characteristics, such as type, intensity, onset, duration, or frequency. In prior studies, the most frequent type of headache reported after TBI was migraine [13, 25]. Up to 49% of post-traumatic headache met the criteria for migraine, followed by tension-type headache (up to 40%) [13]. Although little is known about the natural history of headache after TBI, it has been reported that TBI-attributed headache was persistent over five years or longer after TBI [12, 14, 26, 27]. Some studies reported that new or worse headache prevalence remained consistent with at least 33% of patients [14]. Therefore, our results may have been different if we had evaluated headache characteristics. However, we were only concerned with a subjective measure of headache intensity (NRS), as our focus was on headaches after TBI in general and their potential effects on daily activities. Finally, we did not obtain sufficient information about headache medications. Analgesics were given to all included patients. However, medication may affect headache improvement or may provoke medication-overuse headache [14]. Approximately half of people with headache on 15 or more days per month for more than 3 months have medication-overuse headache [14]. It is very difficult to analyze when or how medications were used. However, we attempted to evaluate the correlation between previously diagnosed headache treated with medication and headache improvement after TBI.

## Conclusions

The course of headache in patients with mild TBI continuously improved until 36-months follow-up. However, 13.1% of patients for whom post-traumatic seizure and traumatic intracranial hemorrhage might be risk factors still suffered from moderate-to-severe headache at 36-month follow-up.

## Abbreviations

CI: Confidence interval
CT: Computed tomography
GCS: Glasgow Coma Scale
LOC: Loss of consciousness
MRI: Magnetic resonance image
mRS: Modified Rankin Scale
NRS: Numeric rating scale
TBI: Traumatic brain injury
